# Cell and Gene Therapies for Mucopolysaccharidoses: Base Editing and Therapeutic Delivery to the CNS

**DOI:** 10.3390/diseases7030047

**Published:** 2019-06-26

**Authors:** Chloe L. Christensen, Rhea E. Ashmead, Francis Y. M. Choy

**Affiliations:** Department of Biology, Centre for Biomedical Research, University of Victoria, 3800 Finnerty Rd., Victoria, BC V8P 5C2, Canada

**Keywords:** mucopolysaccharidosis, base editing, therapeutic, central nervous system, clustered regularly interspaced short palindromic repeats, gene therapies, lysosomal disease, blood-brain barrier, molecular Trojan horse, ultrasound-mediated blood-brain barrier disruption

## Abstract

Although individually uncommon, rare diseases collectively account for a considerable proportion of disease impact worldwide. A group of rare genetic diseases called the mucopolysaccharidoses (MPSs) are characterized by accumulation of partially degraded glycosaminoglycans cellularly. MPS results in varied systemic symptoms and in some forms of the disease, neurodegeneration. Lack of treatment options for MPS with neurological involvement necessitates new avenues of therapeutic investigation. Cell and gene therapies provide putative alternatives and when coupled with genome editing technologies may provide long term or curative treatment. Clustered regularly interspaced short palindromic repeats (CRISPR)-based genome editing technology and, more recently, advances in genome editing research, have allowed for the addition of base editors to the repertoire of CRISPR-based editing tools. The latest versions of base editors are highly efficient on-targeting deoxyribonucleic acid (DNA) editors. Here, we describe a number of putative guide ribonucleic acid (RNA) designs for precision correction of known causative mutations for 10 of the MPSs. In this review, we discuss advances in base editing technologies and current techniques for delivery of cell and gene therapies to the site of global degeneration in patients with severe neurological forms of MPS, the central nervous system, including ultrasound-mediated blood-brain barrier disruption.

## 1. Introduction

The mucopolysaccharidoses (MPSs) are a subgroup of lysosomal diseases (LDs) characterized by the progressive accumulation of partially degraded glycosaminoglycans (GAGs) due to a lack of functional lysosomal enzymes. GAGs are present ubiquitously in connective tissue and play important roles in cell growth and proliferation [1], cell surface binding [2], and histamine storage [3]. When GAG turnover is dysregulated, numerous systems are affected [4]. MPS is characterized by multiple organ involvement of varying severity, affecting the skeleton, joints, skin, eyes and ears, and in some cases the liver, spleen, heart, and central nervous system (CNS) [5]. Patients with mild or attenuated MPS disease can live into adulthood; however, some severe forms of MPS are lethal [5]. All MPS disorders result from inherited autosomal recessive mutations, save Hunter syndrome (MPS II), which is X-linked recessive [4]. The prevalence of MPS as a group is approximately 1:25,000 births [6]. None of these diseases are currently curable, but some patients benefit somatically from enzyme replacement therapy (ERT) through the reduction of GAG build up; however, ERTs fail to cross the blood brain barrier (BBB) and are therefore unsuitable for the treatment of MPS diseases with CNS involvement. Although life-changing for many patients, ERTs are prohibitively expensive with Naglazyme and Elaprase being two of the most expensive drugs to ever be marketed at more than $350,000/year (CDN) [7,8]. Additionally, ERT requires repeated treatments and effectiveness wanes with time since last treatment [9,10]. Cell and gene therapies are proposed to overcome the lack of therapeutic options for patients with MPS disease. Clustered regularly interspaced short palindromic repeats (CRISPR)/CRISPR-associated protein 9 (Cas9) genome editing and a more recent derivative, base editing, offer novel solutions to MPS disease at the genetic level [11]. Base editors for MPS disease and putative in vivo delivery methodologies for these tools, including cell penetrating peptides (CPPs), ultrasound-mediated blood-brain barrier disruption (US BBBD), and nanoparticle (NP) delivery systems, will be discussed at length throughout this review.

### Cell and Gene Therapies for MPS

Cell and gene therapies are recognized options for treatment of MPS disease, wherein whole cells or nucleic acids are delivered in vivo to elicit therapeutic effect. For both, the goal for MPS treatment is to introduce an exogenous gene or cells capable of increasing affected lysosomal enzymes to therapeutic levels. Heterozygous carriers of MPS mutations are asymptomatic and required enzyme thresholds for certain MPS disease are low. For example, MPS I and II patients have residual enzyme levels of <5% [12,13,14] and patients with severe Hurler syndrome (MPS I) have less than 0.13% of normal α-l-iduronidase activity. Therapeutic benefit can be achieved by increasing affected enzyme levels to 0.4–1% in these patients [15]. Additionally, lysosomal enzymes are released into extracellular fluid and endocytosed by neighboring cells through a process called cross-correction [16]. Cross-correction increases the potential therapeutic benefit of cell and gene therapies for MPS by providing existing diseased patient cells with lysosomal enzyme that exceeds residual disease thresholds [16].

Cell therapies may result in a reduction of disease-related symptoms in patients with MPS and are arguably more effective than ERT [17], especially when performed early [18,19,20]. Hematopoietic stem cell transplantation (HSCT), which has been suggested as a treatment for MPS I, II, IV, and VI [19], may result in reduction of neurologic symptoms in patients with CNS involvement [18,19,20]. However, contradictory findings report not only improvement [21] or stabilization [17] of neurologic symptoms but also deterioration post-HSCT [22]. Allogeneic HSCT is recommended as a primary treatment for patients with MPS I [23,24] and MPS II [25,26], but has been debated within the field as a suitable treatment for MPS II [27]. Case studies have reported efficacy of HSCT in MPS IV [28], MPS VI [29], and MPS VII [30]. Issues of allogeneic HSCT include difficulties identifying suitable donors and risks of developing an immune response, i.e., graft-versus-host disease. Although HSCT has been reported to reduce systemic and neurologic symptoms in MPS II, this technique does not completely mitigate disease symptoms, even when initiated in infancy [27]. Additionally, combination cell and gene therapies have been proposed as MPS treatments [31]. Recently, proof-of-concept research using cell and gene therapy has shown efficacy in mouse models for MPS IIIB disease alleviation [32]. In this study, the gene encoding *N*-acetyl-glucosaminidase (NAGLU) was delivered to HSCTs under a myeloid-specific CD11b promoter, which were then transplanted in MPS IIIB mice. Researchers found that treated mice had behavioral correction, increased production of NAGLU, and reduced lysosomal storage of heparan sulfate [32].

Aforementioned caveats of current treatments, in addition to cell and gene therapy delivery limitations introduced by the presence of the BBB, calls for alternative, innovative methods for treating MPS. Important discoveries in the field of stem cell biology have led to the routine adoption of cell reprogramming techniques to generate patient-derived induced pluripotent stem cells (iPSCs) [33]. Combination cell and gene therapies utilizing iPSCs differentiated to neural progenitors may overcome these caveats through intracerebral autotransplantation [34]. In addition to cross-correction of existing neurons, intracerebral transplantation could repopulate glial cells, which may support diseased neurons through improving neuronal connectivity, re-myelination, and removal of cellular waste [35]. As genome editing techniques continue to advance, progenitor cells for autotransplant can be engineered to produce active lysosomal enzymes endogenously. Furthermore, recent advances in delivery techniques will allow for genome editing components to cross the BBB for direct genetic modification in patient’s existing neurons and glial cells.

Remarkable advancements in genome editing technologies have been established since the introduction of CRISPR/Cas9 as a new tool capable of precision genomic alterations in 2012 [36]. CRISPR-Cas9 utilizes a Cas9 endonuclease capable of creating a double-stranded break (DSB) via two catalytically active domains: HNH, which cleaves the target strand, and RuvC, which cleaves the non-target strand [37]. In order to home in on a specific gene of interest at a target location, Cas9 requires a ‘guide’ in the form of ribonucleic acid (RNA) molecule(s) (guide RNA; gRNA). This gRNA is comprised of two regions: the trans-activating CRISPR RNA (tracrRNA; invariable sequence), which interacts at the 3′ end with a binding pocket. The CRISPR RNA (crRNA) sequence is modified to a complementary 20 nucleotides (nts) of the target site. The crRNA interaction with the gDNA target site creates an R-loop and allows for accessibility of Cas9 to cleave both strands of the deoxyribonucleic acid (DNA) backbone at a predictable location between the third and fourth base upstream from a protospacer adjacent motif (PAM), 5′-NGG-3′ [37]. Modifications to the HNH and/or RuvC domains of Cas9 allow for Cas9 nickases (nCas9) or dead Cas9 (dCas9), capable of cleaving only a single strand or incapable of cleaving DNA altogether, respectively. Applications of nCas9 and dCas9 will be discussed later. This PAM sequence, along with the complement to the crRNA, acts as Cas9’s recognition sequence. Although there are many gDNA target sites that are proximal to a 5′-NGG-3′ PAM site, some genomic targets of interest are relatively G/C poor, thus limiting the number of putative target sites with a 5′-NGG-3′ PAM. However, other Cas proteins have since been identified to target a range of alternative PAM sites. For example, Cpf1 (Cas12a; [38]) and FnCpf1 [39] are enzymes that identify T-rich PAM sequences, 5′-TTTV-3′ (where V = A, C, or G) or 5′-TTN-3′, respectively, thus further increasing the number of putative targets to include A/T rich regions. Adding to the repertoire of Cas variations, a group in 2016 bioengineered a 5′-YG-3′ -recognizing Cas protein (where Y = pyrimidine), thus increasing target availability further [40]. Novel genome editing techniques and delivery methods for MPS will be discussed at length throughout this review.

## 2. Genome Editing as a Treatment for Genetic Disease

Precise DNA targeting capabilities of CRISPR-based systems allows for double-stranded cleavage in a gene of interest. One of two repair pathways are employed to repair the resulting double stranded break (DSB): Non-homologous end joining (NHEJ) or homology directed repair (HDR). NHEJ is separated into two possible break fix mechanisms, namely, canonical NHEJ (c-NHEJ) or microhomology-mediated NHEJ [41]. CRISPR/Cas9 elicits specific sequence disruptions easily through NHEJ. Introduction of indels with high efficiency and predictability is valuable for disease modeling through creation of knock-out animals and cell lines. Additionally, NHEJ can abolish existing splice sites, leading to alternate splicing and exon skipping. Long et al. took advantage of NHEJ-induced exon skipping in the gene implicated in Duchenne muscular dystrophy (DMD), *DMD*, in vitro as a proof-of-concept myoediting technique with potential implications as a therapeutic [42]. DMD results from mutations in *DMD*, which encodes dystrophin [43]. The majority of pathogenic *DMD* mutations are harbored in exons 2–10 and 45–55 [43]. Designing individual gRNAs for the nearly 1000 known pathogenic mutations in *DMD* would present an insurmountable logistical hurdle [44], especially when considering clinical applications. Instead, whole exons can be selected for skipping by disrupting intron/exon splice sites, thus removing the pathogenic mutant exons and thereby restoring the majority of dystrophin’s function [42]. Long et al. observed successful myoediting in iPSCs, which conserved exon skipping throughout differentiation to cardiomyocytes [42]. Additionally, they showed that dystrophin function was restored in three-dimensional heart tissue [42].

DNA damage is a common occurrence in normal cellular conditions and chromosomal damage can be a serious issue for cells during replication [45]. Although small sequence errors are introduced, NHEJ is the most predominant repair pathway [45,46]. To overcome this, efficient, error-free biological repair mechanisms exist, namely HDR. HDR requires a template with sequence similarity to direct end ligation and allows for incorporation of precise changes inherent to the challenging DNA template, hereafter referred to as a correction template [45]. Under normal cellular conditions, a homologous chromatid is used as an endogenous template for HDR [47]. This has been exploited in a groundbreaking study where editing of disease-causing mutations in *MYCPB3*, the gene implicated in hypertrophic cardiomyopathy, was observed by microinjection of Cas9-ribonucleoprotein to M-phase human embryos [47]. The HDR pathway can be exploited to create specific sequence alterations by providing an exogenous template containing the desired sequence change, ranging from a single base change to exons and whole genes [48]. Exogenous correction template configuration varies depending on desired edit outcome, cell type, and delivery method. Variations of correction templates include single stranded vs. double stranded oligonucleotides and plasmids, length, and symmetry about the DSB [48,49,50,51]; the correction template composition will depend on the desired outcome and target site composition. One resounding finding in the field is that desired editing efficiency steeply declines when the intended DNA change is more than 10 bp away from the DSB site [51,52,53]. Recently, evidence for a p53 response leading to apoptosis in targeted cells in response to DSBs has raised concern for (1) low editing efficiency and (2) selective propagation of cells with abnormalities in the p53 pathway, thus raising questions regarding clinical translation of genome editing methods that introduce DSBs [54]. Methods that utilize a mutated version of Cas9, called Cas9 nickases, have been proposed to overcome this aforementioned issue, and will be discussed.

### 2.1. Genome Editing for MPS

Due to severe disease progression and limited treatment options for MPS with neurological involvement, we [55] and others [56] have suggested the use of genome editing techniques to aid in the treatment of MPS. Significant progress has been made towards treatments for MPS I and II through zinc finger nuclease (ZFN)-mediated knock-in of *IDUA* and *IDS* in hepatocytes [57] and mouse models [57,58]. Adeno-associated viral (AAV)-mediated delivery of ZFN machinery resulted in the introduction of *IDS* or *IDUA* to a safe harbor locus under a highly expressed promoter and has shown a marked decrease in GAG levels and an increase in enzyme activity, some of which may even reach the CNS based on behavioral measurements in these mouse models [57,58]. Phase 1 clinical trials using ZFNs for insertion of *IDUA* and *IDS* to the albumin safe harbor locus have begun, showing promising preliminary results [59,60]. However, AAVs pose potential development of tumorigenesis due to random insertion and require immunosuppressive drugs, as previously discussed [61,62,63]. Other genome editing methods and delivery methods are currently being investigated as alternative options to AAV gene therapies. CRISPR/Cas9 genome editing has been shown to correct MPS I mutations in human fibroblasts [64] and mice [65] using an alternative to AAV delivery, liposome transfection. Although CRISPR/Cas9 is an exciting option for genome editing in MPS, it is not without its limitations.

### 2.2. CRISPR Caveats

Multiple limitations of the CRISPR/Cas9 system remain to be overcome in order to increase the safety and efficacy of this technology. Three caveats discussed here are (1) issues of off-target effects, (2) the p53 apoptotic response to DSBs, and (3) low efficiency editing in non-cancer cell lines and in disease states that impact cellular repair mechanisms. Although presented as a precise genome editing system, CRISPR/Cas9 has been shown to modify unintended genomic sites, called off-targets. The recognition sequence length for Cas9 is determined by the gRNA length and PAM sequence, approximately 21–23 bases [66]. Given the size of the human genome is 3.2 × 10^9^ bp and assuming each base occurs at a frequency of 1/4, the frequency of a 21–23 base sequence occurring twice in the genome is highly improbable (7.3 × 10^−4^ to 4.5 × 10^−5^; Equation (A1), Appendix A). However, Cas9 recognizes mismatch sequences with up to five bp total mismatches or two consecutive bp mismatches if located at the PAM-distal 5’ end of the crRNA [66,67]. This effectively increases the likelihood that off-target sites may be unintentionally modified. As a common practice, off-target sites are heavily vetted using in silico programs to (1) avoid gRNAs that have a high likelihood of off-targeting, and to (2) determine genomic locations to be screened for off-target effects post-editing [68]. In doing so, a reduction in the negative effects of off-targeting, such as disease-causing alterations in essential genes or tumor suppressor gene knockouts, can be achieved [69].

It was recently determined that non-cancer cell lines fail to incorporate edits at an appreciable rate due to p53-induced apoptosis post-DNA damage (i.e., DSB introduction) [54]. Therefore, cells that have an inactivated p53 pathway are preferentially selected for HDR in genome editing experiments, thus leaving a large proportion of gene modified cells as p53 mutants [54]. Although this finding is troublesome for downstream cell therapies due to the possibility of tumorigenesis post-transplantation, the reversible suppression of the p53 pathway using small molecule inhibitors during genome editing may offer a possible solution [54,70]. These findings may partly explain why cancer cell lines, such as HeLa and HEK293T, show much higher genome editing efficiencies than primary cells and iPS cell lines [48,71,72]. Some genetic diseases may impact genome editing success in vivo by hampering repair pathways, such as an impaired Fanconi anemia (FA) repair pathway in FA patients that has been shown to be an integral pathway in Cas9-induced single-stranded template repair [73]. A number of additional variables that debilitate editing success include: (i) low incidence (0.5–20%) of HDR, even in the presence of a correction template [74,75], (ii) manipulated cells are restricted to late S or G2 phase of the cell cycle [47,76,77,78], and (iii) generated DSBs trigger cellular apoptosis [79]. These factors taken together make HDR a highly inefficient method for mutation correction. A novel genome editing technique, base editing, has been proposed to overcome these issues [11,80,81]. Throughout this review, we will discuss the mechanism of base editing and its application for MPS, as well as putative delivery mechanisms for genome editing systems in vivo.

## 3. Base Editing

### 3.1. Cytidine Base Editors

Base editors are precise tools that mediate the conversion of adenine (A) to guanine (G) or of cytosine (C) to thymine (T) through the use of catalytically defective Cas9 (or other Cas-related proteins) attached to specific DNA deaminases. With the intent of avoiding the complications of HDR, Komor et al. modified the CRISPR/Cas9 system by fusing a cytidine deaminase to a dead Cas9 (catalytically inactive; dCas9) in order to mitigate double stranded DNA cleavage and the necessity of a correction template [11]. This pursuit resulted in the development of the first base editors [11].

**Figure 1:**
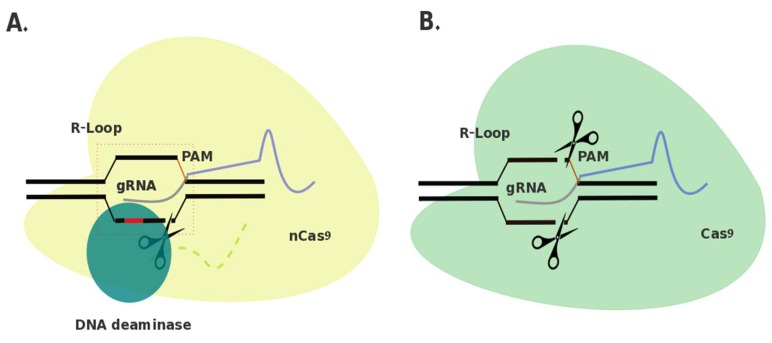
Comparison of base editing and clustered regularly interspaced short palindromic repeats (CRISPR)/CRISPR-associated protein 9 (Cas9) genome editing. **A.** Depiction of a base editor. Cas9 nickase (Cas9^D10A^; yellow) is attached through a linker (light green) to a DNA deaminase. (blue). Guide ribonucleic acid (RNA) (guide RNA (gRNA); purple/grey) guides nCas9 to site of interest, invading the double-stranded (ds) deoxyribonucleic acid (DNA) and forming an R-loop. nCas9 cleaves the target strand 3–4 bp upstream from the protospacer adjacent motif (PAM) site (orange). DNA deaminase is capable of deaminating DNA within a 5-nt window 13–17 nt upstream from the PAM site. **B.** Depiction of CRISPR/Cas9 genome editor. Cas9 (green) is guided to a site of interest via a gRNA (purple/grey). An R-loop is formed through invasion of the dsDNA by the gRNA and Cas9 cleaves the target strand 3–4 bp upstream from the PAM site (orange).

Elimination of the phosphodiester bond hydrolysis capacity of Cas9 allows instead for utilization of the Cas9-RNP complex’s DNA targeting properties, without introduction of indels at the target site. By additionally attaching a cytidine deaminase protein to dCas9 through a linker, this group hypothesized that T > C (or A > G in the complementary strand) single nucleotide polymorphisms (SNPs) could be predictably and efficiently targeted for correction in comparison to using HDR methods with CRISPR/Cas9 and a correction template [11]. Deamination of cytosine results in uracil, which in turn is recognized as thymine by DNA polymerase during replication, and thus reversion from C > T (or G > A) at that position [82]. A gRNA binds complementary to the target sequence upstream of a 5′-NGG-3′ PAM site and an R-loop is formed in the DNA backbone through interaction with Cas9 (or other dCas9 and variations) [83]. This R-loop formation creates a 5-nt activity window at DNA positions 4 through 8 (the 5′ end of the gRNA is complementary to base 1) (Figure 1) [11].

The first base editor version, BE1, was comprised of a rat APOBEC1 cytidine deaminase bound to dCas9 through an XTEN linker and resulted in a C > T editing efficiency of 44% when introduced to target ssDNA in vitro [11]. When introduced to human cells, this efficiency was reduced to 0.8–7.7%, likely due to cellular uracil DNA glycosylase (UDG) recognizing U:G heteroduplexes during DNA repair response [11]. Later versions, BE3 and BE4, incorporate UDG inhibitors and reintroduce Cas9 catalytic activity within the HNH domain (nCas9; Cas9^D10A^), resulting in a notable editing efficiency increase beyond 50% [11,82].

### 3.2. Adenine Base Editors

SNPs constitute nearly two-thirds of all pathogenic or likely pathogenic alterations in the human genome (62,665/98,756 [44]). The majority of SNPs in the human genome, however, are C > T transition mutations due to the prevalent deamination that occurs at 5′-methyl cytosine [84]. These SNPs are not amenable by cytidine deaminase base editors. Gaudelli et al. sought to create a second type of base editor that instead employs an adenine deaminase [80], which would allow for all transition mutations to be potential targets for correction. After seven generations of protein engineering, adenine base editor (ABE)-7.10 was capable of editing six mammalian genomic locations with 58 +/− 4% efficiency [80]. ABE7.10 and BE4 were recently improved upon by introduction of a N- and C-terminal nuclear localization signals (NLS), among other modifications, resulting in ABEmax and BE4max with a 7.1- and a 1.3-fold increase in on-target base editing, respectively [85]. All of the base editors discussed thus far use a Cas9 with a PAM in the form of 5′-NGG-3′. However, other Cas-variations that recognize a multitude of PAM sequences exist, including a PAM 5′-YG-3′-recognizing Cas protein, thus increasing target availability [40]. Another type II CRISPR system uses a Cpf1 [38] of FnCpf1 [39] enzyme that identifies a T-rich PAM sequence, 5′-TTTV-3′ or 5′-TTN-3′, respectively, thus further increasing the number of putative targets to include A/T rich regions. The cytidine deaminase, APOBEC1, was engineered to link to Cpf1 in order to accommodate for these targets [86]. Recently, mouse models for androgen insensitivity syndrome and syndactyly have been generated using ABEs and SaBE3s (a CBE) [87] and correction of the common β-thalassemia *HBB* −28 (A > G) mutation in human embryos using ABEs [88]. These initial studies highlight the utility of base editors in mammalian cells for mutation repair [87,88] and mutagenesis [89], and indicate a potential advantage for mutation correction in other genetic diseases lacking suitable treatments, such as the MPSs. In a recent study, ABEs employing dCas9 were found to increase genome editing efficiency after 10 days in comparison to ABE versions utilizing nCas9 (~8% vs. ~1% target nucleotide conversion; day 3 = ~4% vs. ~5%) [90]. It is postulated that aberrant nicking results in chromosome instability, leading to a loss of targeted cells over time [90].

### 3.3. Base Editing for MPS Diseases

**Figure 2:**
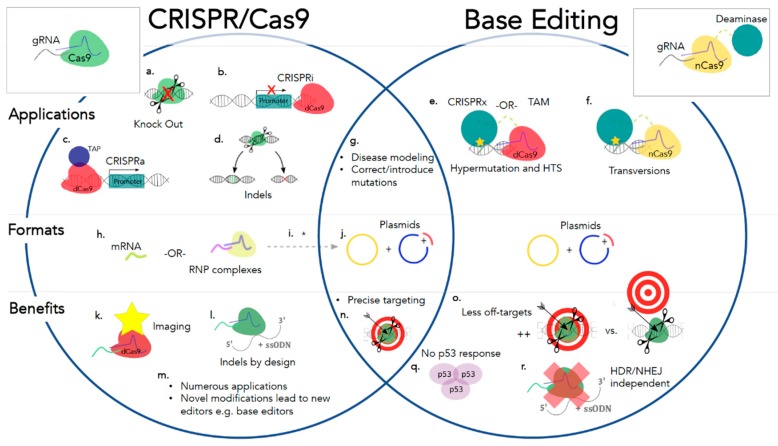
A comparison of the CRISPR/Cas9 and base editing systems on the basis of applications, formats, and benefits. Applications for CRISPR/Cas9 and base editing (a.–g.). CRISPR/Cas9 genome editing can be used to create double-stranded breaks, followed by the employment of non-homologous end joining (NHEJ) for gene knockouts through the introduction of indels (a.) or precise large insertions or deletions through the use of homology directed repair (HDR) and a correction template (d.). Using a dead Cas9 (dCas9), CRISPR interference (CRISPRi) [96] or CRISPR activation (CRISPRa) [97] to introduce a transcriptional activator protein (TAP) for recognition by RNA polymerase and activation at a promoter site (c.), or to bind downstream of a promoter thereby blocking RNA polymerase (b.), respectively. Base editing applications include CRISPRx or targeted AID-mediated mutagenesis (TAM) [98] (e.), which utilizes a dCas9 and deaminase moiety for hypermutation applications and high throughput screening. Precise transversion mutations are realized through cytidine and adenine base editors [11,80] (f.) Both CRISPR/Cas9 and base editing systems are capable of assisting in the creation of cell lines and mouse models for disease modeling and can be used to introduce or correct mutations (g.) Formats for CRISPR/Cas9 and base editing (h.–j.). Formats for CRISPR/Cas9 genome editing include ribonucleoprotein (RNP) complexes, mRNA, or plasmids encoding the necessary components (h.–j.). Base editors are currently available as plasmids but may become available in mRNA or RNP complex formats in the future*. Benefits for CRISPR/Cas9 and base editing (k.–r.). Additional benefits of CRISPR/Cas9 genome editing include gene imaging [99] (k.) and the creation of precise insertions (i.), among numerous other previously mentioned applications. Modifications to the CRISPR/Cas9 system continue to bring about novel methods, such as the base editing system (m.). Base editors show less off-targets [80] (o.), a diminished p53 response [54] (q.), and are independent of cellular repair systems [80] (r.), HDR and NHEJ, in comparison to its CRISPR/Cas9 counterpart. Both systems are highly precise with regards to on-targeting (n.).

**Figure 3:**
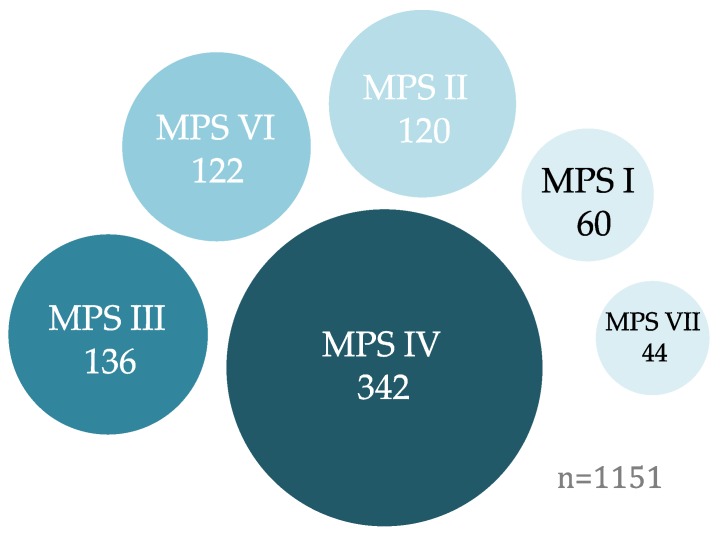
Graphical representation of mucopolysaccharidosis (MPS) mutations as targets for cytidine base editors (CBEs) or adenine base editors (ABEs). A small majority (57%) of known MPS disease-causing mutations (*n* = 1151) are currently targetable with existing ABE and CBE systems [44].

A number of attributes are required for mutation correction using base editing, including (i) a need for a transition mutation, (ii) presence of a PAM sequence within 17 to 13 bases from the target, and (iii) a lack of unintended targets within the activity window (e.g., presence of multiple Cs; Table 1, Figure 2). According to the ClinVar database, 77% of genetic alterations that result in or likely result in MPS disease pathogenesis are SNPs [44]. Given the recent development of ABEmax and BE4max base editors, all MPS-related SNPs that are generated by transition mutations are putative targets for correction. Editing of MPS-related SNPs in patient cells could be used for disease modeling and as a potential therapeutic for MPS. A small majority (57%) of SNPs resulting in MPS have been identified as amenable targets using either CBEs or ABEs (see Table 2, Figure 3) [44]. A number of mutations in *IDUA*, which cause MPS I (Hurler, Scheie, and Hurler-Scheie syndromes), are apt targets for base editors. For example, the c. 1293 G > A nonsense mutation, which results in a stop codon in place of tryptophan in exon 9, and the IVS5AS G > A mutation in intron 5, are targetable by the ABEmax system (Table 2, Table S1). Although this iteration of base editor has not yet been bioengineered, a dFnCpf1 fusion with BE4max could result in a cytidine base editor with recognition of a 5′-TTN-3′ PAM [86]. BE4max-dFnCpf1 with a 5-nt activity window at bases 8 to 4 would target the c. 1469 T > C mutation in exon 10 of *IDUA*, a mutation previously found in patients exhibiting Scheie and Hurler-Scheie syndromes [91,92]. The c. 1402 C > T mutation in *IDS* leads to a severe phenotype of Hunter (MPS II) syndrome but could be corrected using ABEmax base editing through targeting of the antisense strand [93]. A two-base mutation in Morquio B syndrome (MPS IVB), the c. 851–852 TG > CT has the potential to be corrected through using both BE4max and ABEmax base editors; however, suitable PAM sequences do not exist in this region of exon 8 in the *GLB1* gene [94]. For this mutation, the CRISPR/Cas9 system with a correction template could serve as an alternative option. More examples of putative MPS targets for base editing can be found in Table 2.

### 3.4. Pitfalls to Base Editors

Although recent modifications have allowed for new iterations of base editors that allow a large number of mutations to be targeted, activity windows are restricted by the presence of PAM sites in the target region. Given the possible PAM sequences recognized by current and future base editors (Table 1), an increase of putative targets will fall within range of the activity window as research into these tools progresses. Changes in gRNA length may also exhibit a larger proportion of potential edit sites. Furthermore, PAM sites could be created using a step-wise method, where necessary. In the future, advances through protein engineering of base editors may allow for targeting of transversion mutations in addition to transition mutations, further increasing the number of target sites for base editors. When using catalytically active Cas9 and a correction template, it is often suggested that multiple guide sequences are trialed to determine targeting efficiency. However, the limited size of the base editing activity window (5 nt) restricts the number of possible guide sequences and in many cases only a single putative sequence exists. Additionally, recent investigations have led to the identification of transcriptome-wide off-target effects through RNA cytosine deamination [112]. The implications of these transcriptome-wide off-targets are still not fully understood [112]. Ever advancing technologies will lend solutions to these pitfalls, however. For example, Grünewald et al. screened 16 modified BE3 and identified two (BE3-R33A and BE3-R33A/K34A) variants that elicit on-target DNA deamination with comparable efficiencies to BE3 but exhibit a greatly reduced RNA off-targeting [112]. These two BE3 variants have been dubbed SElective Curbing of Unwanted RNA Editing (SECURE) variants and will further increase the specificity and accuracy of base editing systems going forward [112]. In addition, base editors are currently available commercially in only plasmid form. Delivery methods in vitro and in vivo will require optimization prior to therapeutic use and the latter will be discussed further here.

## 4. Delivery of Base Editors to the CNS for Treatment of Neuronopathic MPS

Delivery is an important aspect to consider when designing therapeutics for disorders with CNS involvement. A study conducted in 2000 found that only 1% of all drugs are active in the CNS for diseases other than affective disorders [113,114]. This is largely due to the restrictive nature of the BBB, which encompasses and protects the CNS. Approximately 100% of large pharmaceutical molecules and 98% of small molecules are unable to cross the BBB and enter the CNS [114]; only a small number of pharmaceutical molecules are able to exploit lipid-mediated free diffusion to cross the BBB. Here, we will provide a deeper overview of the barriers impeding CNS drug delivery and suggest potential approaches to navigate these barriers and their applicability for delivery of base editors for the treatment of MPS with CNS involvement.

### 4.1. Barriers for CNS Drug Delivery and Distribution

**Figure 4:**
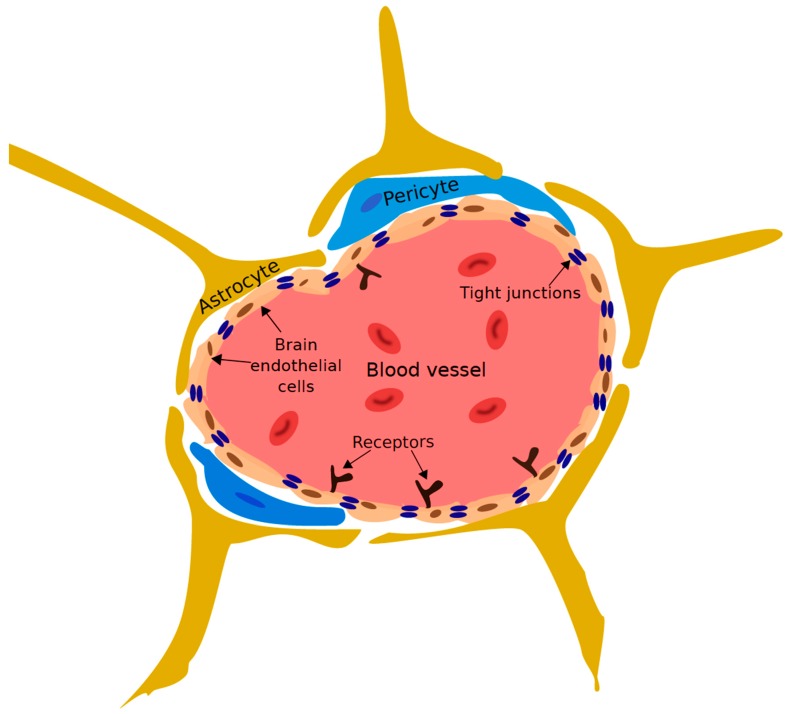
Diagram of the blood-brain barrier (BBB). Brain endothelial cells (BECs) connected by continuous tight junctions line the blood vessels of the cerebrovasculature. Astrocytes, pericytes and other members of neurovascular unit interact with the BECs and support the development and maintenance of the BBB (adapted from [123]).

The vast capillary network that makes up the BBB tightly regulates the passage of ions, molecules, and cells between the blood and the brain (Figure 4) [115,116,117]. This regulation precisely controls the CNS environment, ensuring that it is suitable for proper synaptic transmission and overall neuronal functioning. The primary component of the BBB is brain endothelial cells (BECs) [118]. BECs are distinct from endothelial cells of different tissues: as they lack fenestrations, undergo extremely low rates of transcytosis, and have continuous intracellular tight junctions [117,119]. These features limit the movement of molecules between the blood and the brain via the paracellular and transcellular pathways. To enter the brain via the BBB, molecules must interact with specific transporter receptors expressed on the luminal and abluminal side of BECs [120]. Other elements of the neurovascular unit (NVU), such as pericytes [121,122], astrocytes [123], and neural progenitor cells [124], play key roles in inducing and supporting the BBB. This barrier is essential for the protection and maintenance of the brain environment; however, it is also a formidable obstacle for the delivery of therapeutics to the brain.

Another often overlooked barrier for drug delivery to the brain is the brain parenchyma. The major mechanism of transport throughout the brain parenchyma is diffusion. This is extremely effective at small distances (nm-μm), such as between synaptic clefts or cell bodies; however, for larger distance (mm), diffusion is often slow or entirely restricted. The brain parenchyma is very densely packed with only 20% of total brain tissue volume being extracellular space [125,126]. The estimated diameter of extracellular space between cells is approximately 10–20 nm. Furthermore, the extracellular matrix of the brain is decorated by various electrostatically charged substances, including chondroitin sulfate and heparan sulfate proteoglycans, hyaluronan, and various glycoproteins. These factors greatly restrict the distribution of therapeutic agents by inducing non-specific interactions as well as limiting the geometry and increasing the interstitial viscosity of the ECS. As a result, a large number of therapeutic agents, especially larger macromolecules, exhibit low rates of diffusion and decreased delivery to larger target regions upon direct administration to the CNS [126,127]. Therefore, it is important that the nature of the brain parenchyma as well as the BBB be considered when designing and developing approaches to deliver therapeutic agents to the CNS.

For several decades, approaches to delivering therapeutics across the CNS have focused on mechanically bypassing the BBB, often by direct injection or infusion of the therapeutic into the brain parenchyma, ventricles, or spinal canal. Intrathecal (IT) administration involves mechanically bypassing the BBB and infusing therapeutics into the cerebrospinal fluid (CSF) of the spinal canal or subarachnoid space typically via lumbar puncture or cisterna magna injection. Preclinical studies using IT administration of ERT have been conducted and shown efficacious in several animal models [128,129,130,131,132,133]. Recently, phase I/II clinical trials were completed for IT administration of heparan-*N*-sulfatase (rhHNS) in patients with MPS IIIA using an implantable intrathecal drug delivery device (IDDD) [134]. The results indicated that this approach in general was safe and well tolerated but there were high rates of IDDD malfunctions, suggesting a need to improve IT administration. Although this study was predominantly focused on the safety of IT administrations, it also reported a reduction in heparan sulfate levels in the CSF. These promising results prompted the extension of this study for a period of eight years, during which long term safety and neurocognitive effects of this drug delivery approach will be assessed [135]. This delivery approach is invasive, requiring hospitalization, surgical intervention, and general anesthetic, all of which are costly and come with their own inherent risk. This review will focus on two alternative, non-invasive approaches that could be applied to the delivery base editor machinery or corrected cells to the CNS for the treatment of neuronopathic MPS: ultrasound-mediated (US) BBB disruption (BBBD) and exploitation of native BBB machinery.

### 4.2. Ultrasound-Mediated Blood-Brain Barrier Disruption

BBBD has been investigated as a mechanism of CNS drug delivery since the 1970s. In 1972, Rapoport et al. demonstrated that an intra-arterial infusion of hypotonic solution was capable of temporarily increasing the permeability of the BBB in a rabbit [136]. This led to the development of osmotic BBBD as a technique to deliver substances across the BBB. Several clinical trials have reported promising results for the treatment of malignant brain tumors when osmotic BBBD is used in conjunction with chemotherapy. In a multicenter study published in 2000 used this technique to treat 221 patients with various types of malignant brain tumors and found high rates of stable disease and tumor response with low complication rates [137]. Despite favorable clinical outcomes, there has not been a widespread adoption of osmotic BBBD for the delivery of therapeutics to the CNS. Osmotic BBBD is a highly invasive technique, requiring surgical intervention and general anesthetic [138,139]. These procedures are costly and come with their own inherent risk that are exacerbated by therapeutics that require repeated BBBD.

In the last several years, US technology has generated renewed interest in BBBD as a non-invasive approach to temporarily breach the BBB. Advancements in technology, such as the creation of hemispherical US arrays and application of CT scan and MRI, has facilitated the use of US through the human skull without the need for surgical craniotomy [140,141,142]. In addition, the application of US with microbubble contrast agent has made US BBBD more predictable and safer [143]. Prior to this, the degree of BBB opening using US alone ranged from temporary opening of tight junctions to gross hemorrhage [144]. Microbubbles improve US BBBD by concentrating the acoustic energy of the US in the blood vessel, reducing power required to open the BBB below the threshold necessary for tissue damage [143,145,146]. The primary hypothesis of BBB opening is that the mechanical forces generated by the stable cavitation of the oscillating microbubbles disrupt tight junction between brain endothelial cells, increasing paracellular transport [147]. There is evidence suggesting that this also induces pore formation and endocytosis, leading to increased transcellular transport as well [148,149].

US in conjunction with microbubble contrast agents have been used extensively in preclinical models to open the BBB in various animals, including rabbits [143,150], mice [151,152], rats [153,154], and primates [155,156], as well as being used to deliver various therapeutic agents, including chemotherapy drugs [154], antibodies [157,158], and gene vectors [159,160]. Positive reports regarding safety, tolerability, and effectiveness in these preclinical models have led to clinical translation in the last five years. Phase I/II clinical studies have begun with several neurological diseases, including Alzheimer’s disease, recurrent glioblastoma, and amyotrophic lateral sclerosis [161,162,163,164,165].

#### 4.2.1. Limitations of Ultrasound-Mediated Blood-Brain Barrier Disruption

At present, US BBBD is only effective at opening small regions of the BBB, which limits its application for more diffuse CNS pathologies, like MPS. In the last five years, there has also been growing interest into using unfocused pulses of US for the disruption of larger brain volumes and increased therapeutic biodistribution within the CNS. In particular, the CarThera Research team at the Brain and Spine Institute in France have investigated the use of unfocused US using a SonoCloud^®^ device, which is an implantable US emitter. The advantage of implanting an US device within the skull is that it can eliminate difficulties associated with transcranial US, such as beam distortion, and make repeated disruptions less technically challenging so that it may be possible to perform in a clinic setting [155]. Extensive preclinical studies in rabbits [166] and non-human primates [155,167], have led to phase 1/2 clinical trials for patients with recurrent glioblastoma [162]. The interim results for these clinical trials, indicated that unfocused US BBBD achieved a greater brain volume sonication than previously reported with non-human primate trials and was well tolerated in all patients [168]. Nevertheless, the sonicated brain volume was still limited to only 5 cm^3^, which was not sufficient to reach the entirety of brain tumor tissue. These researchers acknowledged that, in the absence of toxicity, future redesigns of SonoCloud^®^ device may permit for greater brain volume disruption.

Increasing the number of US treatment spots may be a potential approach to increase the volume of disruption. A preclinical study in 2017 by Hsu et al. utilized a one- or two-spot treatment scheme of transcranial weakly focused US with microbubbles to admit intravenously injected α-l-iduronidase (rhIDUA) into the left side of the brain of MPS I mice [169]. They found that one-spot and two-spot resulted in 2.75-fold and 7.81-fold higher levels of IDUA activity, respectively, than the untreated side. This indicated that the two-spot treatment was more efficient at delivering recombinant enzyme to CNS. However, there was also an increase in concentration of albumin in the two-spot (4.224 μg/g tissue) compared to the one-spot treatment (2.512 μg/g tissue) in the brain. Although this level of albumin was not considered neurotoxic, further preclinical studies, especially in larger brain models, would need to be conducted to determine the safety and effectiveness of this approach for increasing brain sonication.

#### 4.2.2. Delivery of Cell- and Gene-Mediated Therapies Using US BBBD

Non-invasive transplantation of corrected neuronal stem cells is a potential application of US BBBD for the treatment of MPS. As mentioned previously, one of the downstream clinical applications for genome editing therapies is autologous transplantation of corrected neural stem cells into the brain of the patient using intracerebral transplantation. Intracerebral transplantation, however, is an invasive procedure with many associated risks, including tissue damage, infection, and edema [170]. Burgess et al. used MRI-guided focused US in rats to deliver iron-filled, GFP-expressing neuronal stem cells across the BBB [170]. MRI confirmed that these cells crossed the BBB following US BBBD. Furthermore, expression of nestin and polysialic acid as well as doublecortin confirmed their survival 24 h following transplantation and ability to differentiate into neurons. At present, a crucial limitation to this approach is the low number of cells that are transplanted in the CNS: only approximately 0.025–0.03% of the injected dose of cells [170,171]. In comparison, the estimated number of cells that survive intracerebral transplantation is on average 5–10% of the injected dose of cells [172]. However, there is ongoing research to enhance stem cell delivery efficiency using focused US in combination with superparamagnetic NPs. Iron oxide loaded stem cells and external magnets could be used to guide cells towards the site of BBB opening after US BBBD thereby increasing delivery across the BBB [171]. If efficiency of delivery can be enhanced in the future, then US BBBD may become a more attractive prospect compared to intracerebral transplantation for the delivery of patient corrected neural stem cells to the brain for the treatment of neuronopathic MPS.

US BBBD could also be used to directly deliver base editing machinery to the CNS for in vivo genome editing in the CNS for patients with neuronopathic MPS. Thus far, base editing technology is still in its infancy and in vivo base editing has not been extensively studied; however, there have been numerous in vivo studies with CRISPR/Cas9 genome editing technology [173]. The majority of these preclinical and clinical studies have focused on delivering CRISPR/Cas9 systemically. However, US BBBD in combination with NP technology may facilitate delivery of base editing machinery across the BBB for correction of the CNS in vivo.

Based on the National Cancer Institute definition, NPs are structures that are less than 100 nm in size; however, this review will define NPs as any sub-micron drug-carrying vehicle. NPs can be composed of natural or synthetic polymers and therapeutic substances can be covalently attached to their surface or encapsulated within them. In recent years, NPs have garnered increasing interest as vehicles for genome editing therapies (for reviews see [174,175]). In comparison to viral delivery systems, NPs have a reduced risk of immunogenicity and genome incorporation, which is important for translating into clinical trials. NPs also have an increased cargo capacity, which is advantageous because the majority of genome editing machinery exceeds the typical cargo capacity for viral delivery systems. Several studies have employed NPs to deliver CRISPR/Cas-9 in vivo. One such group used gold NPs to simultaneously bind and deliver Cas9 RNPs and donor DNA in mice resulted in HDR, a technique coined CRISPR-Gold [176]. CRISPR-Gold uses a cationic polymer to trigger endosomal disruption, allowing for the release of CRISPR cargo into the cytoplasm [164]. CRISPR-Gold shows a high HDR efficiency in iPSCs, embryonic stem cells, and bone marrow-derived dendritic cells of between 3–4%, and in mice of 5.4%, exhibiting the potential of this technique as a non-viral delivery method in a number of cellular contexts both in vitro and in vivo [176]. Gold is convenient due to its low cytotoxicity and ability to complex with a number of organic molecules, including nucleic acids [177]. Similarly, research on zwitterionic amino lipid NP delivery of Cas9-mRNA and gRNA molecules has shown wide somatic distribution of genome editing-mediated knock-in events and may be suitable as a CNS targeting platform when coupled with US BBBD [173]. Although the abovementioned NP designs exhibit reduced cytotoxicity and increased delivery efficiency in comparison to other non-viral methods, such as lipofection, they still have reduced gene delivery efficiency in comparison to viral particles.

In preclinical studies, NPs in the form of microbubbles or conjugated to microbubbles have been employed to deliver therapeutics across the BBB using US BBBD. In 2012, Ting et al. demonstrated that loading 1,3-bis(2-chloroethyl)-1-nitrosourea (BCNU), a chemotherapy agent, into pegylated microbubbles significantly increased the circulatory half-life and reduced cytotoxic liver accumulation of BCNU [153]. When US was used to open the BBB in rats, these functionalized microbubbles also had enhanced delivery of BCNU to the CNS and consequently increased anti-tumor efficacy compared to non-encapsulated BCNU with microbubbles. However, microbubbles are limited in terms of drug loading capacity; therefore, several studies have also explored conjugating other NPs to the surface microbubbles [178,179]. Lin et al. bound glial cell line-derived neurotrophic factor gene carrying liposomes to microbubbles and demonstrated improved gene delivery efficiency to the CNS following US BBBD in mice [159]. These preclinical studies provide a foundation for applying ultrasound-mediated blood-brain barrier disruption (US-BBBD) and functionalized microbubbles as an approach to deliver genome editing therapies in vivo across the BBB to the CNS.

### 4.3. Exploitation of Native BBB Transport Machinery

Another promising approach for CNS drug delivery is exploiting the native transport machinery that exists within the BBB to transport therapeutic agents from the blood into the brain. An advantage of this approach, in comparison to US BBBD, is the potential for increased biodistribution. BBB transport machinery is present throughout the capillary system that perfuses the CNS; therefore, exploiting this transport system would allow for a therapeutic to be distributed globally. This review focuses on two mechanisms for native BBB transport: absorptive mediated transcytosis (AMT) and receptor-mediated transcytosis (RMT), as well as the related techniques that utilize these mechanisms, which could be used to transport gene therapies across the BBB for treatment of CNS pathologies, like MPS.

AMT results from electrostatic and other non-specific interactions and involves a combination of endocytic pathways, including micropinocytosis, clathrin-, and caveolin-mediated endocytosis. CPPs are short peptides less than 30 amino acids in length that vary in charge, polarity, and structure [180]. Their commonality is their ability to exploit AMT to cross biological membranes, including the BBB. The precise pathway of uptake for CPPs is highly dependent on the sequence and concentration of the CPP [181], the type and differentiation status of the receiving cell [182,183], as well as the type of cargo attached to the CPP [184]. This translocation capability of CPPs was first observed in 1988 by Frankel and Pabo, when they noted the Trans-activator of transcription (Tat) domain in human immunodeficiency virus possessed the ability to cross cell membranes [185]. Following this discovery, over a thousand unique CPP sequences have been discovered, leading to a CPP database being established in 2012. Penetratin (RQIKIWFQNRRMKWKK), which was isolated from the antennapedia homeodomain and Tat (CGRKKKRRQRRRPPQC) are the most well characterized and popular CPPs under investigation for BBB delivery. In preclinical trials, these CPPs have been fused to various cargo, including chemotherapy agents [186,187], proteins [188,189], and NPs [190,191,192,193], and facilitated their transport across the BBB.

RMT involves the transport of large molecules across the BBB following interactions with specific receptors. This transcellular uptake pathway transports molecules that are vital for CNS function, including hormones, growth factors, enzymes, and plasma proteins, from the blood into the brain [194]. Receptors for this pathway are highly expressed on the luminal side of BECs. The approach that uses this pathway for the delivery of therapeutics has been coined the molecular Trojan horse (MTH) method. Specific ligands, modified ligands, and antibodies can be fused to therapeutics and used to target RMT receptors on the BBB, which enable the therapeutic to be ferried across the BBB. At present, the most common target receptors for MTHs are the insulin (IR) and transferrin receptor (TfR). These two receptors are found ubiquitously within the human body, with TfR being highly expressed in the BBB, neurons, reticulocytes, and lungs. This approach has been used to transport biologics across the BBB (for review see [195]). Ulbrich et al. exploited the MTH method using human serum albumin NPs with covalently bound insulin or IR mAb to deliver a representative drug, loperamide, to mice through measurement of a significant antinociceptive response increase [196]. Similarly, Zhang and colleagues demonstrated the efficacy of IR mAb PEGylated immunoliposomes for transcytosis of plasmids across the BBB in rhesus monkeys [197]. Both of these studies exemplify the utility of RMT using MTH in mammals. The first MTH, monoclonal antibody against IR, has entered clinical trials for the delivery of an ERT for MPS across the BBB and preliminary data indicate that this therapeutic is well tolerated by patients [198]. Additionally, researchers have demonstrated through phase I/II clinical trials that IDS fused with the anti-human transferrin receptor antibody is safe in humans and significantly reduces heparan sulfate and dermatan sulfate in CSF, demonstrating successful crossing of the BBB [199].

RMT and AMT are also effective tools for BBB transcytosis when coupled together. CPP-Tf liposomes utilizing the CPP poly-l-arginine (PR) have demonstrated a 2-fold increase in BBB penetration when compared to single ligand liposomes in rats; however, PR is predicted to negatively impact cell viability thus reducing practical utility [200]. This study led to an investigation into alternative biocompatible CPPs, where Tat and Penetratin CPP-Tf liposome varieties were identified as efficient and biocompatible options for the delivery of doxorubicin in vitro and in vivo [200]. Similarly, Zong and colleagues created dual-functionalized Tat-T7-liposomes and determined that doxorubicin delivery is synergistically increased in comparison to single ligand liposome counterparts in an in vitro glioblastoma model [201]. Another quantified dual-targeting strategy for BBB drug delivery includes the use of magnetic PLGA NPs with T7 modification targeting TfR, resulting in a five-fold increase in delivery in a glioma mouse model [202]. Given these findings, exploitation of IR and TfR and dual functionalization that exploits RMT and AMT is an attractive prospective for biocompatible drug delivery in vivo.

#### 4.3.1. Delivery of Gene Therapies Across Using RMT and AMT

In addition to therapeutic drugs, liposomes and NPs loaded with gene therapeutics have also shown efficacy via RMT and AMT pathways. For example, reduction of amyloid-beta plaques in a mouse model of Alzheimer’s disease (AD) was achieved via the delivery of a gene and peptide co-delivery [203]. This was actualized via the assembly of PEGylated and rabies virus glycoprotein 29 (RVG29)-coated dendrigraft poly-l-lysines (DGLs) in conjunction with BACE1 shRNA-encoding plasmids as NPs [203]. RVG29 binding to n-acetylcholine receptors on brain capillary endothelial cells allows for RMT to the brain. BACE1 shRNA allows for downregulation of amyloid-beta plaques, whereas DGLs inhibit tau-fibril formation, thus demonstrating a two-pronged targeted gene and peptide therapy for AD. In addition to drug cargo, Penetratin-Tf liposomes can also deliver plasmids across the BBB of mice (transgene expression increase of 11.9 ± 1.3%), thus suggesting that non-viral CNS-directed gene therapies may be a suitable alternative to viral delivery [204]. Although a number of studies have demonstrated in vivo genome editing in both neurons and glial cells in mice [205,206], these studies have relied primarily on intracranial injection or viral delivery in order to bypass the brain. Advances towards non-viral and non-invasive systemic genome editing have exhibited successful gene targeting in a number of in vivo models thus far [86,87,207]. Adoption of US-BBBD, RMT, or AMT in conjunction with current leading in vivo genome editing delivery methods, such as CRISPR-Gold [176], will provide a promising area for future investigation in this field.

#### 4.3.2. Limitations of AMT and RMT for Delivery of Gene Therapies Across the BBB

Key limitations of AMT and RMT for CNS-specific gene therapies include systemic mistargeting and limitations of exogenous gene expression. Due to the ubiquitous expression of IR and TfR throughout the body and the nonspecific nature of many CPPs, RMT, and AMT result in nonspecific distribution of payload systemically. Differing AMT or RMT formulations may lead to brain-specific targeting, such as cationic bovine serum albumin PEG-PLGA NPs [208]. Transgene expression post-administration has been a longstanding barrier for non-viral gene therapy [209]; however, advances in genome editing have allowed for the manipulation of endogenous genes in vivo, thus mitigating the need for gene therapies that rely on transgene expression. For non-viral AMT or RMT gene therapies to be viable solutions for patients with MPS, future research should focus on increasing global CNS distribution and successful therapies will likely employ a combination of the abovementioned techniques.

## 5. Conclusions

Limitations in available therapies currently hinder treatment of MPS. Historically, ERT, substrate reduction therapy, and HSCT have offered some patients symptom reduction, but are largely ineffective for MPS with neurologic involvement. Cell and gene-based therapies that exploit novel genome editing technologies, including base editor variations of the CRISPR/Cas9 system, are at the forefront of research into treatments for genetic disease. Suitable methods for delivery of genome edited cells or genome editing machinery to patients still need to be optimized; however, US-BBBD, RMT, and AMT techniques discussed here offer a promising direction to be explored with respect to MPS treatment. Future work will focus on increasing genome editing efficiency and fidelity and enhancing delivery of genome editing cargo to the CNS in vivo.

## Figures and Tables

**Table 1:** Existing and proposed base editors with various protospacer adjacent motif (PAM) sequences and possible base modifications.

Cas Protein.	PAM Sequence (5′- 3′; N = any base, V = A, C, or G)	Existing Engineered Base Editors	Possible Base Modifications
Cas9^D10A^, Cas9, dCas9	NGG	BE4max; ABEmax; dABE; dCBE	C > T; A > G
Cpf1 (Cas12a)	TTTV	BE4max	C > T
FnCpf1	TTN	None	N/A
C2c2	Avoidance of 3′ G [95]	None	N/A

**Table 2:** Example target mucopolysaccharidosis mutations for base editing *.

Disease	Gene	Mutation (Amino Acid Alteration; Codon Δ)	Nucleotide Alteration	Compatible Base Editor (PAM) (N = any base, V = A, C, or G)
MPS I (Hurler, Hurler-Scheie, Scheie)	*IDUA*	p.W402X TGG → TAG [100]	c. 1293 G>A	ABEmax (NGG)
		p.Q70X CAG → TAG [101]	c. 208 C > T	ABEmax (NGG)
	*IDUA*	p.L490P CTG → CCG [102]	c. 1469 T > C	BE4-FnCpf1 (TTN) [86]
MPS II (Hunter)	*IDS*	p.S333L [103]	c. 1122 C > T	ABEmax (NGG)
	*IDS*	p.R468T CGG → TGG [104]	c. 1402 C > T	ABEmax (NGG)
MPS IIIA (Sanfilippo A)	*SGSH*	p.R245H CGC → CAC [105]	c. 746 G > A	ABEmax (NGG)
MPS IIIB (Sanfilippo B)	*NAGLU*	p.E153K GAG > AAG [106]	c. 457 G > A	ABEmax (NGG)
MPS IIIC (Sanfilippo C)	*HGSNAT*	p.R351X	c. 1084 C > T	ABEmax (NGG)
MPS IIID (Sanfilippo D)	*GNS*	p.R355X CGA > TGA [107]	c. 1063 C > T	ABEmax-Cpf1 fusion (TTTV)
MPS IVA (Morquio A)	*GALNS*	p.R386C CGT > TGT [108]	c. 1156 C > T	ABEmax (NGG)
MPS IVB (Morquio B)	*GLB1*	p.W273L [94]	c. 851–852 TG > CT	CRISPR/Cas9
MPS VI (Maroteaux-Lamy)	*ARSB*	p.R95Q CGG > CAG [109]	c.284 G > A	ABEmax (NGG)
MPS VII (Sly Syndrome)	*GUSB*	p.A619V GCG > CTG [110]	c.1856 C > T	ABEmax-Cpf1 fusion (TTTV) [110]

* See Table S1 for base editing window design; Base editors are not currently capable of correcting transversion mutations [111].

## Supplementary Materials

The following are available online at https://www.mdpi.com/2079-9721/7/3/47/s1, Table S1: gRNA designs for MPS mutation targeting.

## Appendix A

Equation (A1):

(A1) $${\left(\frac{1}{4}\right)}^{n}\times \left(3.2\times 10^{9}\right)\;\mathrm{where}\;n=21,\;22,\;\mathrm{or}\;23$$
