# Supplementary material for: Cell and Gene Therapies for Mucopolysaccharidoses: Base Editing and Therapeutic Delivery to the CNS
Source: Diseases. 2019 Jun 26;7(3):47. doi: 10.3390/diseases7030047 (PMC6787741; doi:10.3390/diseases7030047)
Supplement: Supplementary file 1 [file diseases-07-00047-s001.pdf]

Cell and Gene Therapies for Mucopolysaccharidoses: Base Editing and Therapeutic Delivery to the CNS

Chloe L. Christensen, Rhea E. Ashmead and Francis Y.M. Choy *

Table 1. Guide RNA (gRNA) designs for mucopolysaccharidosis (MPS) mutation targeting. Complimentary to Table 2 within main text. gRNA sequence designs for targeting a number of reported mutations causative for MPS disease. gRNA sequences are written in 5′→3′ orientation, base editing (BE) window is underlined, protospacer adjacent motif (PAM) sequences indicated in brackets, and causative mutations are written in red.

Gene	Nucleotide Alteration	Compatible Base Editor (PAM)	gRNA Sequence (5′ → 3′), BE window, (PAM), mutation
IDUA	c. 1293 G>A	ABEmax (NGG)	GCTCTAGGCCGAAGTGTCGC(AGG)
	c. 208 C>T	ABEmax (NGG)	TGAGCTGCTAGTCCCAGCTG(AGG)[*]
IDUA	c. 1469 T>C	BE4-FnCpf1 (TTN)	GGCGCCCGGGCCGGCCCGTC(TTC)
IDS	c. 1122 C>T	ABEmax (NGG)	ATCCAAGGTAAATGCAATGA(TGG)
IDS	c. 1402 C>T	ABEmax (NGG)	GGAATCAAAAAATGCTTCAGA(AGG)
SGSH	c. 746 G>A	ABEmax (NGG)	GTCGGCCACATGGACCAAGG(TGG)
NAGLU	c. 889 C>T	ABEmax (NGG)	GAGCGAAAGATAGACTGGA(TGG)
HGSNAT	c. 1084 C>T	ABEmax (NGG)	CCCAATCACTGCAGCACACC(AGG)
GNS	c. 1063 C>T	ABEmax-Cpf1fusion[¶] (TTTV)	CCTCAAACCAACAGTGGAAC(TTTG)
GALNS	c. 337 A>T, c. 901G>T, and c. 1156 C>T	None[Δ], None[Δ], ABEmax (NGG)	TCGCCACGATAATAGAAGAT(AGG)[*]
GLB1	c. 851-852 TG>CT [94]	CRISPR/Cas9[†]	(1) ATCAATTCTGAATTCTATAC(TGG)<br>(2) ATACTGGCCTGCTAGATCAC(TGG)<br>(3) ACTGGCCTGCTAGATCACT(GGG)<br>(4) ATGGCCTGCTAGATCACTG(GGG)
ARSB	c.284 G>A	ABEmax (NGG)	TCGCAGAGCCAGCTGCTCAC(TGG)
GUSB	c.1856 C>T	ABEmax-Cpf1 fusion[¶] (TTN) [110]	GGAACACTGCACTTTTTTGG(TTG)

[*]Mutation lies outside of base editing window, but still within region capable of being targeted by adenine deaminase. The first nine nucleotides upstream of the PAM sequence are buried within Cas9, but evidence that the 11th to 18th nucleotides are capable of undergoing deamination [11]. [¶]Currently conceptual. [Δ] Base editors are not currently capable of correcting transversion mutations [111]. [†]Only sense gRNA options creating a cut site within 10 bp of the mutation are shown.
